# Identification of candidate gonadal sex differentiation genes in the chicken embryo using RNA-seq

**DOI:** 10.1186/s12864-015-1886-5

**Published:** 2015-09-16

**Authors:** Katie L. Ayers, Luke S. Lambeth, Nadia M. Davidson, Andrew H. Sinclair, Alicia Oshlack, Craig A. Smith

**Affiliations:** Murdoch Childrens Research Institute, Royal Children’s Hospital, Flemington Road, 3052 Parkville, VIC Australia; Department of Paediatrics, University of Melbourne, Parkville, VIC Australia; Department of Anatomy and Developmental Biology, Monash University, Clayton, VIC 3168 Australia

**Keywords:** Sex determination, Gonad, Sexual differentiation, Chicken embryo, Ovary, Testis, RNA-seq

## Abstract

**Background:**

Despite some advances in recent years, the genetic control of gonadal sex differentiation during embryogenesis is still not completely understood. To identify new candidate genes involved in ovary and testis development, RNA-seq was used to define the transcriptome of embryonic chicken gonads at the onset of sexual differentiation (day 6.0/stage 29).

**Results:**

RNA-seq revealed more than 1000 genes that were transcribed in a sex-biased manner at this early stage of gonadal differentiation. Comparison with undifferentiated gonads revealed that sex biased expression was derived primarily from autosomal rather than sex-linked genes. Gene ontology and pathway analysis indicated that many of these genes encoded proteins involved in extracellular matrix function and cytoskeletal remodelling, as well as tubulogenesis. Several of these genes are novel candidate regulators of gonadal sex differentiation, based on sex-biased expression profiles that are altered following experimental sex reversal. We further characterised three female-biased (ovarian) genes; *calpain-5* (*CAPN5*), *G-protein coupled receptor 56* (*GPR56*), and *FGFR3* (*fibroblast growth factor receptor 3*). Protein expression of these candidates in the developing ovaries suggests that they play an important role in this tissue.

**Conclusions:**

This study provides insight into the earliest steps of vertebrate gonad sex differentiation, and identifies novel candidate genes for ovarian and testicular development.

**Electronic supplementary material:**

The online version of this article (doi:10.1186/s12864-015-1886-5) contains supplementary material, which is available to authorized users.

## Background

Gonadal sex differentiation in vertebrate embryos involves sexually dimorphic gene expression, leading to ovarian or testicular development. While a number of key gonadal differentiation genes have been identified and validated in a variety of organisms, large gaps in our understanding still exist [1, 2]. This likely underlies the significant number of human Disorders of Sex Development (DSD’s) that remain unexplained at the genetic level [1]. Identification of novel regulators of embryonic ovarian and testis differentiation is therefore required, and will greatly improve our understanding of normal and aberrant gonad sexual differentiation. The chicken embryo provides a unique model system for studying gonad development because development occurs *in ovo* (in the egg), allowing gonadal development to be directly manipulated [3]. In chickens and other birds, as in mammals, sex is determined by sex chromosome inheritance. However, birds have a ZZ male: ZW female sex chromosome system, which is the opposite of the XY male: XX female system found in mammals. Genes located on the sex chromosomes drive differentiation of the “bipotential” gonads into ovaries (in ZW females) or testes (in ZZ males). Several key genes involved in mammalian gonadal sex differentiation are conserved in chicken, including male up-regulated *DMRT1*, *SOX9* and *AMH*, and female up-regulated *Aromatase, FOXL2 and WNT4/RSPO1* [2–12]. However, the Y-chromosome linked *SRY* gene, which initiates testis development in mammals, is absent in birds. The best candidate master sex switch in birds is *DMRT1*, which is present on the Z sex chromosome and is expressed more highly in male gonads compared to females prior to and during sexual differentiation. Knockdown and over-expression of *DMRT1* causes feminisation and masculinisation of the gonads respectively [3, 7]. Although a W-linked female (ovarian) determinant may yet exist, our extensive analysis has so far not produced a convincing candidate gene [13, 14].

In chicken embryos, the gonads form on the medioventral surface of the mesonephric kidneys at embryonic day 3 (E3 - Hamburger and Hamilton stage 18) [15]. At this stage, they are undifferentiated or “bipotential”. At E6 (HH29), gonads begin morphological differentiation into testes in ZZ embryos or unilateral ovary in ZW embryos. Sex-specific gonadal morphology emerges at this time, and a small number of genes showing sexually dimorphic expression have been identified, notably the ovarian determinants *FOXL2* and *aromatase* in females [11, 16, 17], and *DMRT1*, *HEMOGEN* and *SOX9* in males [1, 4, 8, 10, 18–21]. However, in chicken, the exact functional relationships among these genes are unclear. Furthermore, human DSD’s imply the existence of other as yet unidentified gonad-determining genes in vertebrates generally.

Previous screens in the mouse embryo have employed various methods to isolate novel genes regulating gonadal sex differentiation, such as high throughput whole mount *in situ* hybridization (WISH) [22], differential display [23, 24], cDNA subtractive hybridization [25–28] cDNA microarrays [19, 29, 30] and RNA-seq on sorted germ cells [31]. However, technical limitations often mean that these approaches assay only a small fraction of the transcriptome, and some studies have sampled stages when the gonads are larger but when differentiation has already occurred. Nevertheless, several of these large-scale studies have successfully identified hundreds of genes with sexually dimorphic expression in embryonic mouse gonads. A microarray study on sorted gonadal somatic cells (using the *Sf1-GFP* reporter) from 10.5 and 11.5dpc found numerous sex-biased genes specific to the early differentiating Sertoli and granulosa cells [32]. Another study used microarrays on gonadal somatic cells to identify 2306 genes expressed in a sex-specific manner prior to, during and after gonadal sex differentiation (E10.5 – E13.5) [33]. An independent microarray screen compared sorted supporting cells (*Sry* or *Sox9* – eGFP), interstitial or stromal cells (*Mafb*-eGFP) and germ cells (*Oct4*-eGFP) from XY and XX mouse gonads at E11.5, E12.5 and E13.5 [34]. These studies as well as the others mentioned above identified novel candidates such as *Aard, Cst9, Mmd2, Cbln4*, some of which have been further characterised in gonad development [24, 35–38]. Whilst these studies represent extensive transcriptome profiling of embryonic gonads, deep RNA sequencing (RNA-seq) is considerably more sensitive [39]. In addition, RNA-seq reveals expression for all mRNAs, not only genes covered by microarray, allowing an unbiased analysis.

In the current study, RNA-seq is used to assess sex-biased gene expression at the onset gonadal sex differentiation in embryonic chicken gonads. Our rationale is based on the assumption that genes important for ovary versus testis development will show sex-biased expression. We previously performed RNA sequencing on E4.5 gonads, at the bi-potential stage prior to sexual differentiation [14]. By comparing gene expression from this early stage to E6 (the onset of morphological differentiation), we find novel genes and pathways that are activated sex-specifically at the time of sexual differentiation; more than 1000 genes are transcribed in a sex-biased manner at this stage, and the majority of these become biased between E4.5 and E6. This is primarily due to an increased expression of autosomal, rather than sex-linked, genes between these time points. Gene ontology and pathway analysis indicate that these genes are involved in extracellular signalling as well as driving morphological changes such as ECM, cytoskeletal remodelling and tubulogenesis. We also find significant enrichment of genes reported in the embryonic mouse gonadal screens in the genes identified in our chicken screen, highlighting conservation of molecular mechanisms underlying gonad differentiation. Several novel candidate gonadal sex differentiation genes are identified and validated *in vivo*. Three female-biased (ovarian) genes are further characterised; *calpain-5* (*CAPN5*), *G-protein coupled receptor 56* (*GPR56*), and *FGFR3* (*fibroblast growth factor receptor 3*). These genes may play an important role during ovarian development. This study provides insight into the earliest steps of gonad sex differentiation, and identifies novel candidate genes for ovarian and testis development.

## Results

### Significant sex-biased gene expression in early differentiating gonads (E6.0/stage 29)

To assess gene expression in the early differentiating testes and ovaries, paired gonads from E6 (HH29) were dissected and mRNA isolated as previously described [14]. For each sex, 2 pools of 18 gonad pairs (from 18 individuals) were collected. Messenger RNA was then processed and sequenced (Methods), resulting in an average of 46 000 000 read pairs per sample. We have previously performed the same analysis on E4.5 paired gonads [14]. Expression for each gene and differential expression (DE) analysis were then calculated (Methods). Gene expression is calculated in Fragments Per Kilobase of Exon Per Million Fragments Mapped (FPKM). Significance is calculated using False Discovery Rate (FDR) (see Methods).

**Fig. 1:**
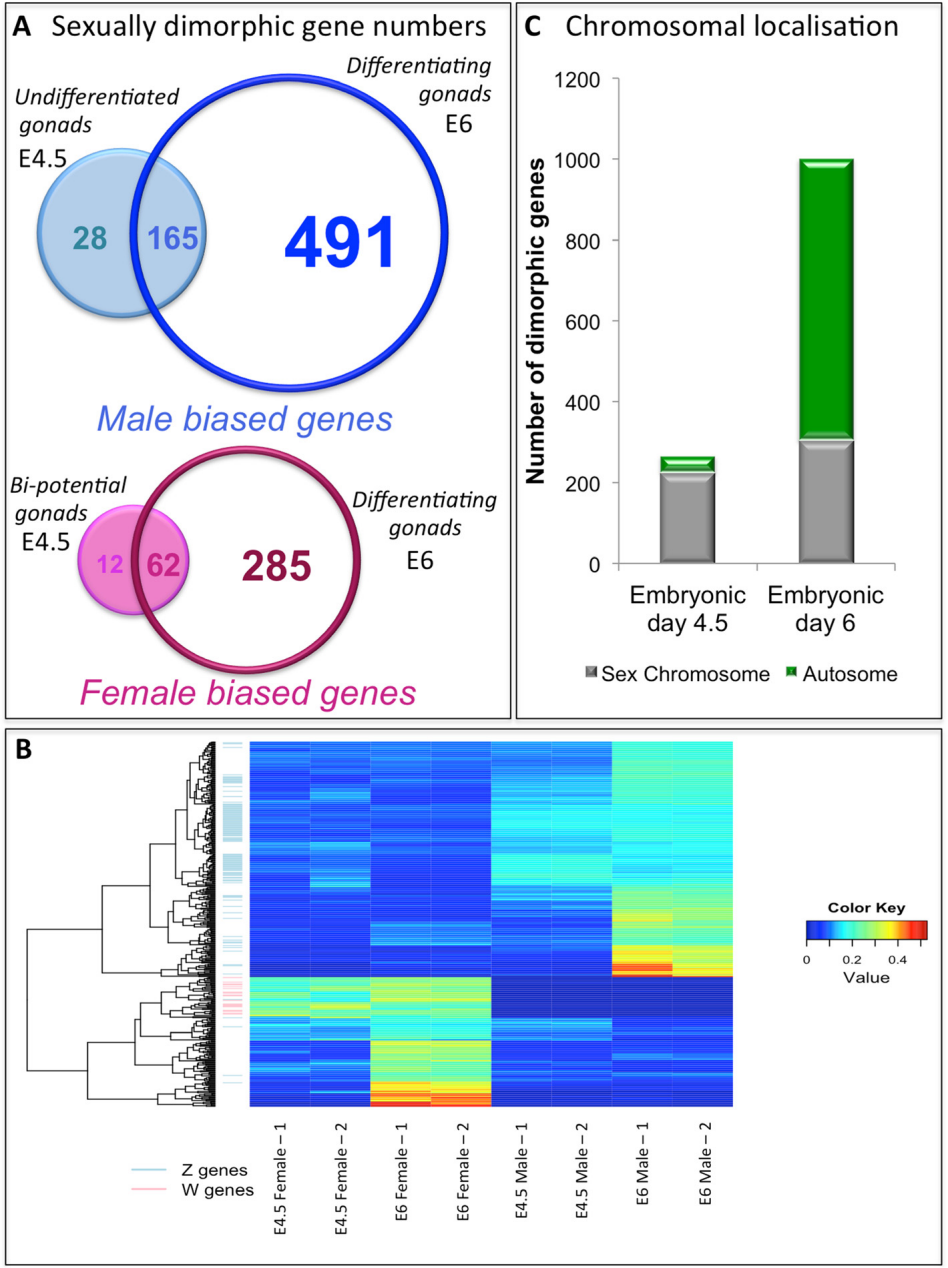
Sexually dimorphic gene expression in the bi-potential and differentiating chicken embryonic gonads. **a** Venn diagrams indicating the number of significantly DE (FDR <0.001) genes for each time-point (undifferentiated gonads E4.5, differentiating gonads E6). This is divided into male-biased (blue) and female-biased (red). 193 genes were male biased at E4.5, and 656 at E6. Of these, 165 genes were DE at both time points, with only 28 genes being DE at only E4.5. 74 genes were DE in a female biased manner at E4.5, and 347 at E6. 62 of these were DE at both time points. **b** A heatmap of the top 400 DE genes for each sample (2 females and 2 males for each time point). The colour scale indicates the proportion of counts in each sample with respect to the sum across samples, after normalising for library size. With red indicating the highest expression relative to other samples and dark blue being the least. On the left is hierarchical clustering of these genes. The known W-linked genes are indicated by pink lines while the Z-linked genes are indicated by blue lines. **c** The number of DE genes (both male and female biased) found on the autosomes (green bars) versus the sex chromosomes (grey bars), for each time point

RNA-seq analysis detected expression of 17108 genes. As the gonads begin to differentiate at E6, a total of 1003 genes showed significant DE between the sexes (FDR <0.001). Of these, 347 were female-biased and 656 were male-biased (Fig. 1a). In contrast, at the earlier time point of E4.5, when the gonads are morphologically undifferentiated, only 267 genes were significantly DE (193 male biased genes and 74 female biased genes; FDR <0.001) (Fig. 1a, b). Thus, between E4.5 and E6 as the gonads begin to differentiate, a four-fold increase in sexually dimorphic transcript number was observed (from 1.56 to 5.86 % of all expressed genes). Substantial overlap was observed between the genes that were sexually DE at E4.5 and those at E6; 165 genes in males and 62 in females (85.5 and 83.8 % of the E4.5 DE genes were also DE at E6.5 in males and females respectively) (Fig. 1a). Indeed, of the 267 DE genes at E4.5, only 40 (15 %) lost this bias at E6 (Fig. 1a) and the majority of these (all but 5) reside on the sex chromosomes [W, Z or suspected W chromosome (un-random)] [14]. This may reflect changes in dosage compensation of these sex chromosome genes or changes in the expression of W linked genes [14, 40–42].

Hierarchical clustering was performed on the 400 most significantly DE genes (those with the lowest *P*-values) from E4.5 and E6 gonads. The clustering of genes on the basis of expression level is represented in a heat map (Fig. 1b). W-linked genes clustered together and showed high differential expression in both female samples at both time-points (Fig. 1b), consistent with our previous observations [14]. Similarly, Z-linked genes clustered together. Importantly, autosomal genes that were DE were primarily present in E6 and not E4.5 gonads, with female DE genes clustering together for each sample and male DE genes clustering. The overall number of DE sex-linked genes did not greatly change between the time-points (E4.5 = 224, E6 = 304). This indicates that the increase in biased genes expression between E4.5 and E6 was largely due to transcriptional changes in autosomal genes, which increased from 43 DE genes at E4.5 (16 %) to 699 (70 %) at E6 (Fig. 1c). Taken together, these data show that between E4.5 and E6, there was a significant increase in the number of autosomal genes showing sexually dimorphic expression. These genes are novel candidates for contributing to gonad sexual differentiation.

### Known sex-differentiation genes show dimorphic expression

The expression profiles of known or putative regulators of gonadal sexual differentiation were assessed at E4.5 and E6 (Table 1). Of the known sexual regulators based on previous studies in chicken or mammals, only one autosomal gene was significantly DE at E4.5 in females; *FOXL2* (FDR <0.001). At E6, *FOXL2* continued to be differentially expressed, and in addition *CYP19A1/Aromatase*, *HSD17B1* and *NROB1* (*DAX-1*) transcripts became female enriched (Table 1). Four genes showed significantly male-biased expression at both time-points; *HSD17B4, DMRT1, HEMOGEN, and AMH* (FDR <0.001)*. Inhibin A and B* trended towards sexual dimorphism (FDR 0.0027, 0.0012 respectively) at E4.5, and by E6 both were significantly male-enriched (Table 1). *SOX9* showed male-biased expression at E6 only, confirming previous studies suggesting that it lies downstream of Z-linked genes *DMRT1* and *HEMOGEN* [3, 20]. These data imply that major components of the sex determination pathways are activated between E4.5 and E6 (developmental stages HH 25/26 to HH 29/30). Several other sex-development genes show gonadal expression but no sexual dimorphism (Table 1). Indeed, many known regulators of bipotential gonad formation such as *LHX9, EMX2, GATA4* and *FOG2* were very highly expressed in both female and male E4.5 gonads, while expression decreased significantly in E6 gonads (Table 1). This confirms that the E4.5 gonads are still undifferentiated, whereas at E6 the differentiation process has commenced, reflected by down-regulation of genes required for the formation of the undifferentiated gonad and up-regulation of sex-differentiation genes.

**Table 1:** Expression of known and putative sex genes in embryonic chicken gonads, revealed by RNA-seq

Gene name	Gene ID	E4.5 gonads				E6 gonads			
		Female	Male	*P*-Value	Fem:Male FC	Female	Male	*P*-Value	Fem:Male FC
**CYP19A1**	**ENSGALG00000013294**	**0.041**	**0.004**	**0.115**	**7.905**	**122.663**	**0.061**	**0.000**	**1977.060**
**FOXL2**	**ENSGALG00000021009**	**5.954**	**0.931**	**0.000**	**6.178**	**43.945**	**0.996**	**0.000**	**44.013**
**HSD17B1**	**ENSGALG00000027429**	**6.805**	**3.736**	**0.010**	**1.756**	**111.132**	**15.557**	**0.000**	**7.106**
**SOX10**	**ENSGALG00000012290**	**4.548**	**2.636**	**0.032**	**1.789**	**3.563**	**1.476**	**0.000**	**2.170**
**NR0B1**	**ENSGALG00000016287**	**14.337**	**12.882**	**0.743**	**1.157**	**39.036**	**16.230**	**0.000**	**2.126**
**BMP2**	**ENSGALG00000008830**	**6.477**	**4.325**	**0.314**	**1.443**	**12.433**	**6.028**	**0.000**	**2.066**
DMRT2	ENSGALG00000026790	0.062	0.083	0.880	0.722	0.117	0.058	0.498	1.998
CBLN4	ENSGALG00000007772	0.737	0.534	0.764	1.311	0.307	0.156	0.189	1.997
FGF9	ENSGALG00000025748	1.911	1.673	0.901	1.097	1.686	1.129	0.074	1.498
RSPO1	ENSGALG00000001946	1.063	0.970	1.000	1.048	0.462	0.324	0.453	1.429
CBX2	ENSGALG00000023338	9.871	10.314	0.870	0.917	6.147	6.185	1.000	0.998
CTNNB1	ENSGALG00000011905	425.969	449.949	0.979	0.977	273.702	246.522	0.977	0.983
LHX9	ENSGALG00000002223	105.310	104.413	0.962	1.038	79.966	76.430	0.680	0.921
EMX2	ENSGALG00000009302	122.967	117.497	0.886	1.090	77.461	75.557	0.742	0.904
NR5A1	ENSGALG00000001080	38.599	50.040	0.129	0.745	52.266	60.456	0.444	0.863
MAP3K4	ENSGALG00000020003	8.796	9.495	0.709	0.885	7.635	9.513	0.133	0.803
ZFPM2/FOG2	ENSGALG00000016078	45.987	47.630	1.923	1.016	0.581	0.788	0.024	0.798
MAP3K1	ENSGALG00000014718	34.143	48.070	0.276	0.671	25.100	33.224	0.323	0.756
WNT4	ENSGALG00000004790	26.562	29.000	0.979	0.956	14.432	17.492	0.104	0.728
GATA4	ENSGALG00000022821	2060.253	2782.683	1.367	0.770	531.061	519.498	0.269	0.726
SOX8	ENSGALG00000005263	1.240	1.502	0.791	0.851	1.207	1.625	0.126	0.655
DMRT3	ENSGALG00000010161	0.905	1.440	0.290	0.647	0.927	1.482	0.022	0.551
**HSD17B4**	**ENSGALG00000002187**	**23.172**	**45.287**	**0.000**	**0.530**	**22.422**	**40.765**	**0.000**	**0.488**
**INHBB**	**ENSGALG00000028770**	**5.751**	**10.439**	**0.001**	**0.532**	**19.171**	**41.431**	**0.000**	**0.462**
**DMRT1**	**ENSGALG00000010160**	**80.634**	**152.988**	**0.000**	**0.548**	**87.845**	**207.079**	**0.000**	**0.373**
**INHA**	**ENSGALG00000011234**	**1.698**	**3.300**	**0.003**	**0.497**	**4.081**	**24.239**	**0.000**	**0.168**
**HEMGN**	**ENSGALG00000023061**	**4.119**	**10.467**	**0.000**	**0.380**	**16.499**	**112.377**	**0.000**	**0.146**
**SOX9**	**ENSGALG00000004386**	**1.608**	**2.130**	**0.635**	**0.785**	**1.073**	**10.613**	**0.000**	**0.090**
**AMH**	**ENSGALG00000024368**	**7.965**	**25.730**	**0.000**	**0.322**	**56.914**	**1878.929**	**0.000**	**0.027**

The expression of known or implicated sex genes in the female and male gonads, at E4.5 and E6. Expression is measured in FPKM (fragments per kilobase of exon per million fragments mapped). *P*-values (FDR) indicate whether the difference between females and males is significant, and Fem:Male fold-change (FC) is the difference between females sample expression and male samples expression. Genes are ranked in terms of their FC at E6. Those in bold are significantly different (*P*-value (FDR) <0.001) between sexes at E6

While no RNA-seq analysis has yet been reported for whole embryonic mouse gonads, large-scale transcriptional screens have been carried out [22]. To assess conservation of sexually dimorphic genes in other organisms, chicken DE genes at E6 (*P*-value <0.05) were compared with those from three separate large-scale mouse microarray screens [32, 33, 43]. The first of these studies assayed somatic cells from XX and XY embryonic mouse gonads at E10.5, E11.5, E12.5 and E13.5, with E11.5 – 12.5 being equivalent to chicken E6 (stage 29/30) [33]. The second study used isolated somatic cells from XX and XY gonads at 10.5 and 11.5dpc and identified genes showing male up-regulation or down-regulation specific to 11.5dpc, as well as female up-regulation at 11.5dpc [32]. In the third study, isolated supporting cells, stromal/interstitials cells and germ cells were profiled from XX and XY mice [34]. All three of these studies used different methods of assessing differential expression, but all used a *P*-value of 0.05 to assess DE genes at each time point. To compare our data to these studies, we used a *P*-value of 0.05 to find E6 DE genes in the chicken. This results in a list of 2123 genes. This gene list was then compared with those identified as sex-biased at 11.5 and 12.5dpc from Nef *et al.* [33] and Jameson *et al.* [34], and with those identified in the other study [32]. Fisher’s exact tests were also carried out to test for statistically significant enrichment of each data set in our set. When we compared each data set to our DE gene list we found significant enrichment (*P*-value <0.001) for each study (Table 2). Our RNA-seq study was carried out on whole gonads, and will hence encompass expression from various populations of cells include supporting cells and germ cells. By comparing to each cell lineage from the Jameson *et al.* study, we could also see that DE genes associated with supporting cell (443), and interstitial cells (210) are represented in our data set at a significant level (Table 2*P*-value <0.001). Germ cells were enriched with a *P*-value of 0.00123 (163 genes). Overall, we found 76 genes in common between the three mouse studies alones (specifically in the supporting cell populations) (Table 2). Of these, 20 genes were also DE in our study (Table 2). Thus significant overlap between differentiating mouse and chickens gonads was observed, suggesting that similar genetic pathways are engaged between mammals and avians, validating the use of chicken embryonic gonads to screen for novel sex determination candidates.

**Table 2:** Genes found in additional mouse gonadal screens

Study		Chicken E6 DE genes in common	*P*-value for enrichment
[1]	Beverdam and Koopman, [32]	89	1.32E-10
[2]	Nef *et al.* [33]	46	4.70E-05
[3]	Jameson *et al.* [34], Supporting cells	443	2.54E-19
[4]	Jameson *et al.* [34], Stromal/interstitial cells	210	5.48E-18
[5]	Jameson *et al.* [34], Germ cells	163	1.23E-03
	Common between [1], [2], [3]	76	N/A
	Common between [1], [2], [3] and chicken E6	20	N/A
		*Col14a1, Cyp11a1, Ogt, Slc20a1, Lect1, Nedd9, Prss12, Fst, Hpse, Gpm6b, Tesc, Atp1a1, Dapk1, Adm, Scarb2, Mef2c, Tyro3, Gdnf, Bace2, Nac1*	

Comparison of chicken genes showing significant DE at E6 (*P*-value < 0.05) and three mouse microarray screens Beverdam *et al.* [32], Nef *et al.* [33] and Jameson *et al.* who assayed supporting cells, germ cells and interstitial/stromal cells [34]. 20 genes were common between our study and the three mouse supporting cell gene sets. These are listed

20 genes were common between all studies of somatic cells and our study (Table 2). These genes were *Col14a1, Cyp11a1, Ogt, Slc20a1, Lect1, Nedd9, Prss12, Fst, Hpse, Gpm6b, Tesc, Atp1a1, Dapk1, Adm, Scarb2, Mef2c, Tyro3, Gdnf, Bace2 and Nac1.* Some of these genes have been implicated in sex development previously. *CYP11A1* mutations cause congenital adrenal insufficiency and with partial or complete 46,XY sex reversal in humans. GDNF or glial cell line derived neurotrophic factor exerts a proliferative effect on developing mouse Sertoli cells [44]. Finally, *Tescalin* (*Tesc*) has been previously described as an early testis gene [45, 46], although no gonadal phenotypes have been described in a null mutant mouse [47]. We also analysed the overlap between these studies and our dataset using a *P*-value cut off of 0.001 to determine DE genes. This dataset has 1003 genes, half of what we have using the less stringent *P*-value of 0.05. Consistently, we found reduced numbers of overlapping genes, but enrichment was still significant between all groups (Additional file 1: Table S3).

### Transcriptional changes in the differentiating gonads reflect morphological differentiation

To elucidate novel pathways engaged during sexual differentiation of ovaries and testes, pathway analysis was performed on the dataset. To this end, genes were filtered that were only sexually dimorphic at E6 (FDR <0.001), but not in the undifferentiated gonads (E4.5). Genes previously implicated in sex determination or differentiation were removed, to reveal novel activated pathways. Gene ontology (GO) analysis was carried out on this list of 774 genes (Additional file 2: Table S1) using DAVID [48, 49]. We assessed three ontological categories: Biological process (BP), Cellular component (CC) and Molecular function (MF) (Table 3). The top 3 BP categories in DE genes were tube development, cell proliferation and respiratory tube development. The CCs most represented were extracellular and cell surface. The MFs were lipid binding, growth factor activity and ATPase activity (Table 3).

**Table 3:** Gene ontology and KEGG pathway analysis of gonad differentiation candidates

Gene ontology category	Gene count	%	*P*-value
Biological process			
Tube development	19	2.7	1.90E-06
Regulation of cell proliferation	30	4.3	1.40E-06
Respiratory tube development	12	1.7	2.20E-05
Cellular component			
Extracellular region	50	7.2	1.20E-05
Cell surface	14	2	9.40E-04
Extracellular region part	28	4	1.60E-03
Molecular Function			
Lipid binding	15	2.1	2.70E-03
Growth factor activity	11	1.6	3.90E-03
ATPase activity	7	1	4.10E-03
KEGG pathway Term	Gene count	%	*P*-value
Focal adhesion	21	3	2.90E-04
Dorso-ventral axis formation	5	0.7	1.30E-02
Regulation of actin cytoskeleton	16	2.3	2.20E-02
ECM-receptor interaction	9	1.3	2.60E-02
Cytokine-cytokine receptor interaction	13	1.9	5.00E-02

Genes showing DE expression at E6 but not at E4.5 were analysed for gene ontology and KEGG pathway enrichment using DAVID software [48, 49]. The top 3 gene ontology categories for biological process, cellular component and molecular function are shown, along with the gene count, percentage of total genes input and the *P*-value. The top 5 KEGG pathways are shown for the same data set

To assess the probable functions of the genes in this data set, we used DAVID functional analysis of KEGG pathways [48]. The top five KEGG pathways that were significantly engaged (based on calculated *P*-value <0.05) were focal adhesion, dorsal-ventral axis formation, regulation of actin cytoskeleton, ECM-receptor interaction, and cytokine-cytokine receptor interaction (Table 3). Numerous DE genes were implicated in each of these pathways (i.e. Additional file 3: Figure S1A, B). The high number of genes involved in extracellular function and in the engagement of pathways involved in actin cytoskeletal remodelling and ECM function is consistent with the significant morphological changes that are taking place as the gonads differentiate into ovaries or testis. Indeed, the embryonic ovary and testis differ significantly in their morphology; the ovary develops a thickened cortex to house germ cells, while the medulla becomes fragmented, and the testis organizes into basement membrane bound seminiferous cords that enclose germ cells and supporting Sertoli cells. These morphological events begin at E6. Vasculature and seminiferous cord development are important in the testis [43], which was reflected here in the number of genes implicated in tube formation and development. Panther pathway analysis also revealed that the TGF-β pathway was significantly represented (*P*-value = 0.05), including BMP2, 3, 4 and 7 and BMPR2 as well as JUN and Myostatin. Indeed, BMP signalling has been implicated in ovarian and follicle development [50–53], although the exact role of these BMPs at this early stage needs to be elucidated.

### Novel candidates for testis and ovarian development

Known sex determination genes were effectively identified from the RNA-seq data set, many of which had sexually dimorphic expression only at E6. Based on this analysis, the sequencing data was interrogated to identify novel genes involved in chicken gonadal sex differentiation. For potential testis-determining genes, the data was filtered for genes showing sexually dimorphic expression at E6 (FDR < 0.001) which were male biased (Log2 fold change/Fem:male ratio of less than 1) and with a male gonadal expression of more than 10 FPKM. FPKM of 10 was chosen as a cut-off based on the expression of known gonad genes and on previous studies in our lab that suggest *in vivo* methods such as WISH do not detect the expression of genes with less than this value (data not shown). These testis genes were ranked according to *P*-value and the top 45 genes were tabulated (Additional file 4: Table S2). This list includes previously mentioned known sex-regulators such as *DMRT1*, *HEMOGEN*, *AMH* and *SOX9*. From this list, four candidate genes were chosen for further study, based on gene ontology and expression. Specifically we chose genes with a variety of expression levels/fold changes, and which are thought to be involved in transcriptional regulation or morphogenesis/cell differentiation. These were *laminin1* (*LAMA1*), *BMP receptor 2* (*BMPR2*), *zinc-finger 385B* (*ZNF385b*) and a novel, uncharacterised gene on the Z chromosome that we designated *NZP* (Table 4). To identify genes involved in ovarian development, genes were filtered for significant sexual dimorphism at E6 (FDR <0.001), female bias (Log2 fold change > than 1) and with a female expression greater than FPKM 10. All W-linked genes were removed as these have been analyzed previously [14], and the remaining genes were ranked according to *P*-value (Additional file 4: Table S2), and the top 45 taken. From this list, three novel candidate genes for ovarian development were chosen based on their expression profiles and on their gene ontology. These were *calpain-5* (*CAPN5*), *G-protein coupled receptor 56* (*GPR56*) and *fibroblast growth factor receptor 3* (*FGFR3*) (Table 4).

**Table 4:** RNA-seq expression of novel candidates for *in vivo* validation

Gene name	Gene ID	Position	E4.5 gonads				E6 gonads			
			Female	Male	*P*-Value	Fem:Male FC	Female	Male	*P*-Value	Fem:Male FC
GPR56	ENSGALG00000001085	chr11:529482-535232	7.796	5.909	0.328	1.277	58.422	6.309	0.000	9.258
FGFR3	ENSGALG00000015708	chr4:82973160-83026374	7.806	6.302	0.659	1.184	14.262	2.479	0.000	5.770
CAPN5	ENSGALG00000000750	chr1:192455766-192506610	13.372	14.865	0.852	0.924	36.491	8.763	0.000	3.690
BMPR-II	ENSGALG00000008459	chr7:11320598-11416297	16.131	26.090	0.396	0.578	9.868	37.448	0.000	0.264
ZNF385B	ENSGALG00000009012	chr7:14343681-14477554	20.765	31.951	0.000	0.624	25.092	74.033	0.000	0.338
LAMA1	ENSGALG00000014615	chr2:99400292-99499147	20.282	24.625	0.337	0.783	10.520	30.161	0.000	0.349
NZP	ENSGALG00000021163	chrZ:42249103-42257660	12.561	34.068	0.000	0.383	22.707	113.353	0.000	0.175

Expression (FPKM) is shown for female and males gonads at E4.5 and E6, with *P*-values (FDR) for the sex differences (DE) and the fold change between sexes

**Fig. 2:**
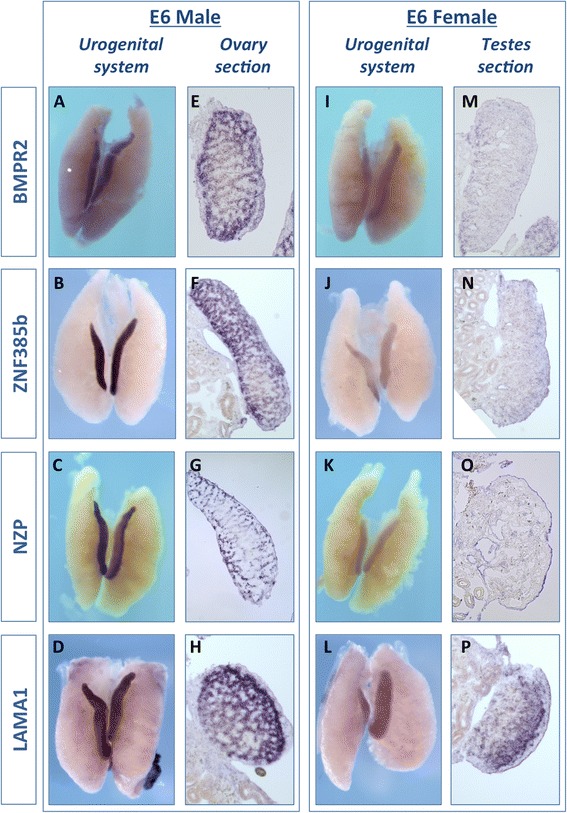
Male candidate gene expression *in vivo.* Whole mount *in situ* hybridisation for 4 male-biased candidate genes, on E6 UGS (UGS) from males and females. *BMPR2* is more highly expressed in males (**a**, **e**) than in females (**i**, **m**). In over-stained sections, *BMPR2* appears to be expressed in the testis cords (**e**). *ZNF385b* shows greater expression in males (**b**, **f**) than females (**j**, **n**), consistent with RNA-seq. In males it is expressed in the cords (**f**). *NZP*, a novel Z-protein, is expressed in males more highly than females (**c**, **g** versus **k**, **o**). It is also expressed in the cords of males (**g**). *LAMA1* is also higher in males (**d**) than females (**l**) and is expressed in testis cords in males (**h**), with some weak expression in the juxta-cortical medulla in females (**p**). These results are consistent with RNA-seq data. Typically, 3 UGS from each sex were used for each probe, and these images are representative. A sense control probe did not show any staining for any of the candidate genes (data not shown)

**Fig. 3:**
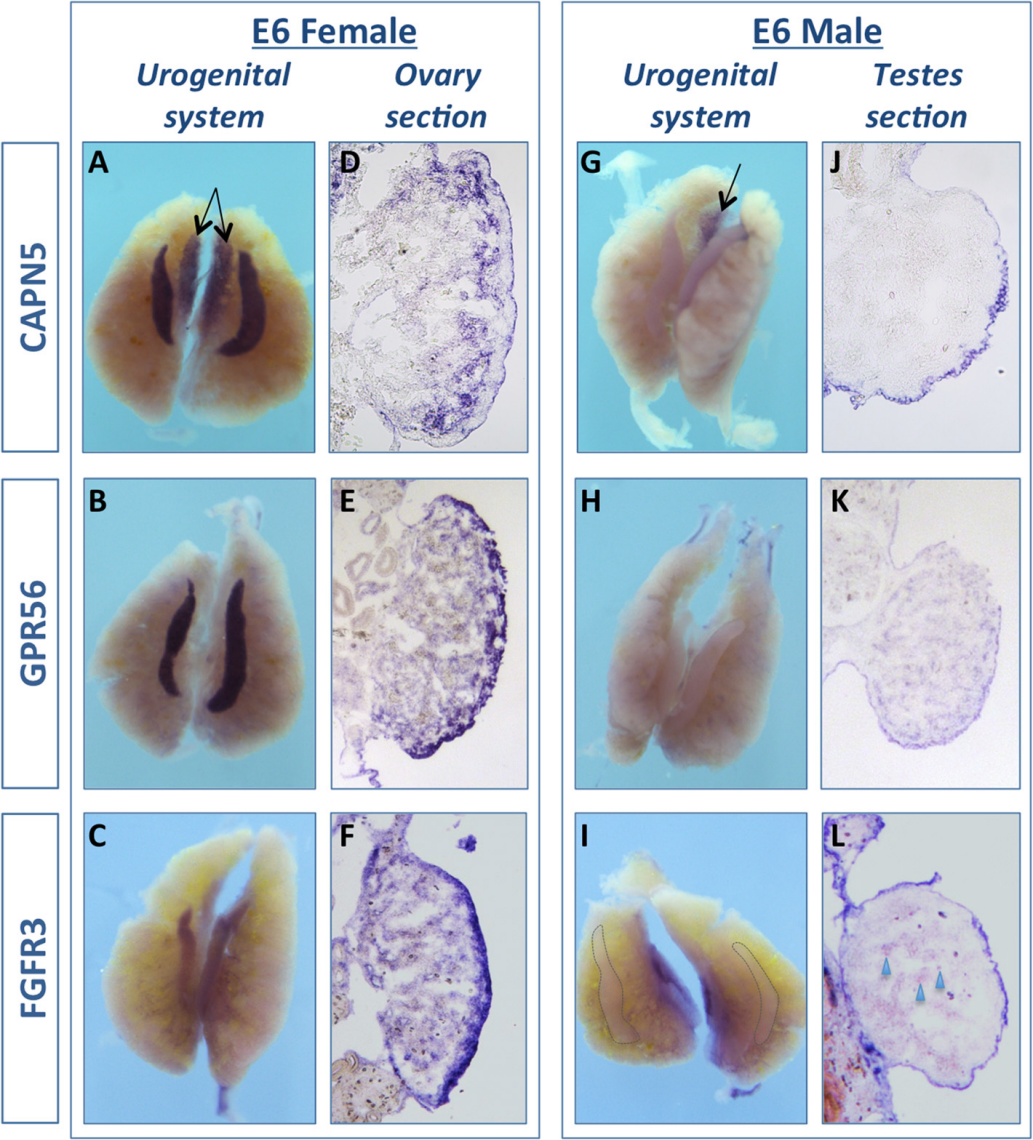
Female candidate genes expression *in vivo.* Whole mount *in situ* hybridisation for 3 female-biased candidate genes, on E6 UGS from males and females. *CAPN5* is more highly expressed in female (**a**) than in males, where only low staining is observed (**g**, **j**). *CAPN5* is also expressed in the adrenal glands in both females and males (**a**, **g** *arrows*). In over-stained sections *CAPN5* appears to be expressed in the juxta-cortical medulla of the ovary (**d**). *GPR56* shows strong expression in female gonads (**b**) and no expression in the males (**h**, **k**), consistent with RNA-seq. In females it is expressed in the cortex of the ovary, with a lower level in the medulla (**e**). *FGFR3* is also higher in females (**c**) than males, which show low expression (**i** – gonads delineated by lines). *FGFR3* is mostly expressed in the ovarian cortex (**f)**, and in the male weakly in a subset of cord cells (**l**, *arrow heads*). These results are consistent with RNA-seq data. Typically, 3 UGS from each sex were used for each probe, and these images are representative of what is seen. A sense control probe is also tested, and did not show any staining for any of the genes (data not shown)

Whole-mount *in situ* hybridisation (WISH) of chicken embryonic urogenital systems (UGS) at E6 was used to confirm mRNA expression of the seven candidate genes (Figs. 2 and 3). By this method, genes could be assessed for their degree of gonad specificity, and their expression could be localised to a gonadal cell type. WISH confirmed male-biased expression in the E6 gonads for all four of the testis candidate genes; *LAMA1*, *BMPR2, ZNF385B,* and *NZP* (Fig. 2a-d versus i-l). Over-staining and sectioning showed that all four of these genes were expressed in the seminiferous cords of the developing testis (Fig. 2e-h). *BMPR2, ZNF385b* and *NZP* had only low level, diffuse expression in the ovary (Fig. 2i-o), while *LAMA1* had expression in the juxta-cortical medulla of the ovary (Fig. 2l, p). None of these genes were notably expressed at other sites within the urogenital system.

All three candidate ovarian genes showed strong female-biased expression at E6.0 (stage 29/30) (Fig. 3a-c versus g-i) in WISH. *CAPN5* and *GPR56* were strongly expressed in female gonads but only weakly expressed in males (Fig. 3a, b and g, h). *GPR56* and *FGFR3* had no expression in other sites within the urogenital system, while *CAPN5* showed expression in the adrenal glands of both males and females (Fig. 3a, g arrows). Sectioning revealed that *CAPN5* was expressed in a subset of medullary cells close to the cortex – the juxta-cortical medulla (Fig. 3d). *GPR56* and *FGFR3* were expressed primarily in the cortex of the ovary (Fig. 3e, f). Taken together these results indicate that the gonad RNA-seq data was robustly validated by *in situ* hybridisation.

### Novel sex-differentiation candidates show altered expression following experimental sex-reversal of gonads

RNA-seq and *WISH* analysis revealed the expression of three female- and four male-biased gonadal genes. To test the involvement of these genes in the gonadal differentiation pathways, experimental sex reversal was carried out using the aromatase inhibitor, fadrozole. Fadrozole inhibits the activity of the aromatase enzyme, blocking oestrogen production, and resulting in female to male sex reversal of the embryonic gonads [54, 55]. Fadrozole was injected into fertile eggs at E3 (stage 19) and gonads were harvested from embryos at E9 (stage 33). *WISH* was used to compare gene expression to that of stage-matched controls.

**Fig. 4:**
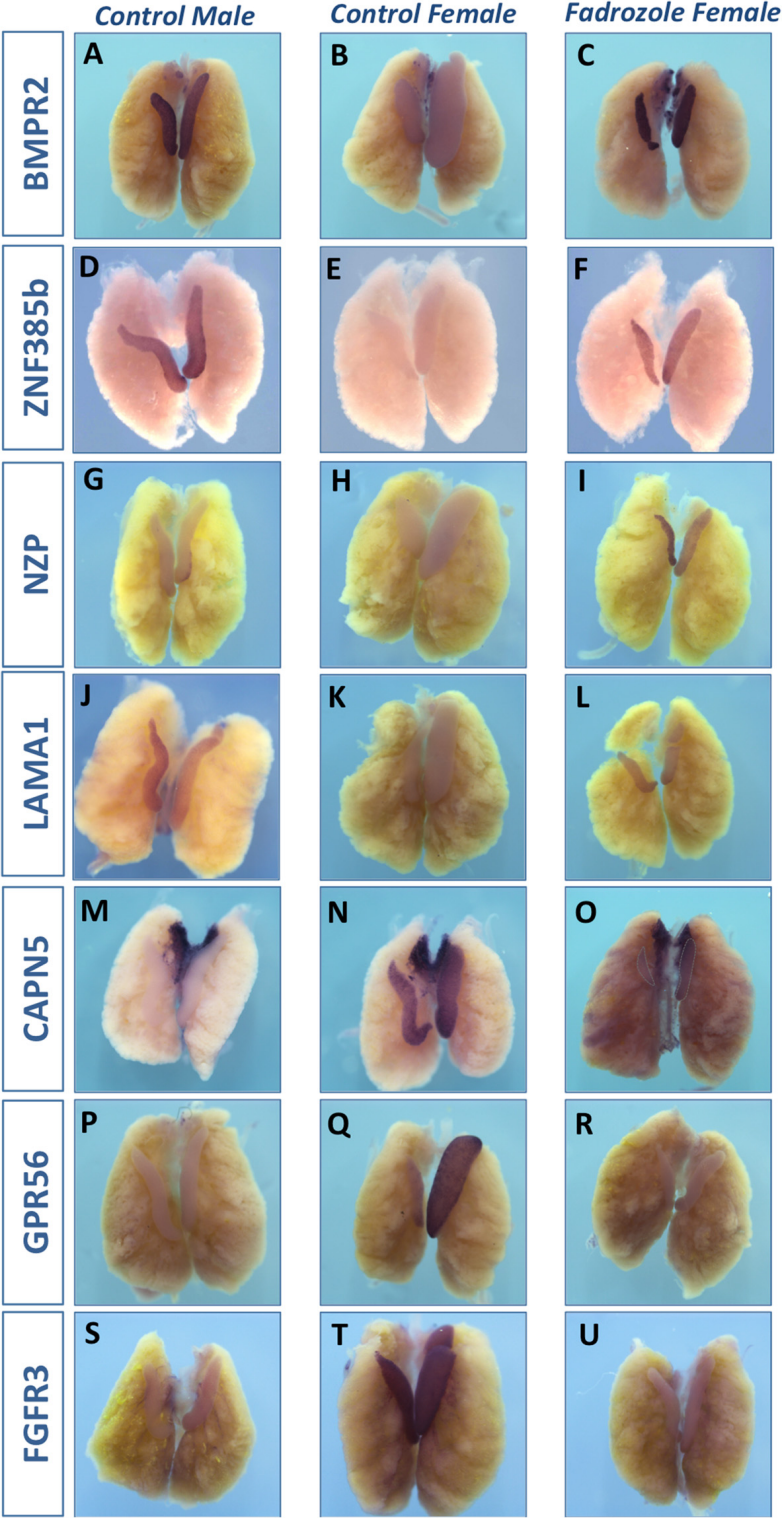
Expression of candidate genes is altered in sex-reversed gonads. Whole mount *in situ* of E9 UGS from male controls, female controls or fadrozole treated females. The expression of male candidate genes *BMPR2* (**a**-**c**) and *ZNF385b* (**d**-**f**) changed dramatically in fadrozole treated embryos (**c**, **f**), where expression was almost at the same level as untreated males (**a**, **d**). *NZP* also had increased expression in treated females (**i**) compared to untreated (**h**), although expression of this gene has generally dropped in control males at this stage (**g**). *LAMA1* expression had also dropped in males at E9 (**j**), and only low expression was found in females (**k**). A slight increase in expression was seen in treated females (**l**). *CAPN5* was lost from the gonads (dotted lines) but not the adrenal gland of treated females (**o**) compared to control females (**n**) . In the gonads it obtained a similar level of expression to control males (**m**). *GPR56, which is absent in males* (**p**), was also lost from treated females (**r**) whereas in untreated females it is strongly expressed in the left ovary (**q**). Similarly, fadrozole treatment abolished *FGFR3* staining in the gonads (**u**), to a similar level as untreated males (**s**) from normal strong expression in females (**t**)

The male-biased genes, *BMPR2* and *ZNF385b* (Fig. 4a-c and d-f) were up-regulated in sex-reversed females (Fig. 4c, f) compared to control females (Fig. 4b, e), suggesting that the expression of these is a consequence of male sex differentiation. While *NZP* also showed up-regulation in the treated female (Fig. 4i), expression of this gene is low at this time-point in both sexes (Fig. 4g, h). Similarly, *LAMA1* expression was weaker in the male at this time point, and only a weak activation was observed in the female treated with fadrozole (Fig. 4j-l). Further functional analysis of these three candidate testis genes is currently underway. Antibodies have been raised against ZNF385b and knockdown is being conducted using our established virally delivered RNAi approach [7]. The female-biased genes *CAPN5* (Fig. 4m-o), *GPR56* (Fig. 4p-r) and *FGFR3* (Fig. 4s-u) all showed down-regulation in fadrozole treated female gonads (Fig. 4o, r, u) compared to the untreated female. In GPR56, only the left gonad of females showed robust expression at this point (Fig. 4q). This was lost in fadrozole treated gonads (Fig. 4r). These data show that these genes are expressed in female-specific cell types as a consequence of the early steps of ovarian differentiation. Their expression is subsequently lost when ovaries are sex reversed. These data indicate that the RNA-seq screen was used to successfully identify candidate genes for ovarian or testis development. As our interest lies in identifying novel genes involved in ovary differentiation, we further focused on *CAPN5, GPR56* and *FGFR3.*

### Candidate ovarian genes are translated during gonad development

Only a small number of genes have so far been shown to play a deterministic role in ovarian differentiation and development [1]. Screens such as the one described here provide additional candidate genes, which will fill gaps in our understanding of ovarian morphogenesis. Therefore we focused on the female-biased candidates *FGFR3*, *CAPN5* and *GPR56*. FGFR3 is a receptor for fibroblast growth factors (FGFs) which have been shown to play a central role in gonad differentiation and germ cell development [56–58]. A role for FGFR3 has not been described in the ovary during embryogenesis. *Calpain-5* (CAPN5) encodes an intracellular calcium-dependent cysteine protease, and has similarity to the *Caenorhabditis elegans* sex determination gene *tra-3*. CAPN5 has not been implicated in gonad development in vertebrates. GPR56 is an adhesion-associated G-protein coupled receptor that mediates intracellular signal transduction. GPR56 null mice have impaired testis formation and infertility [59]. No phenotype has been reported in female mice.

**Fig. 5:**
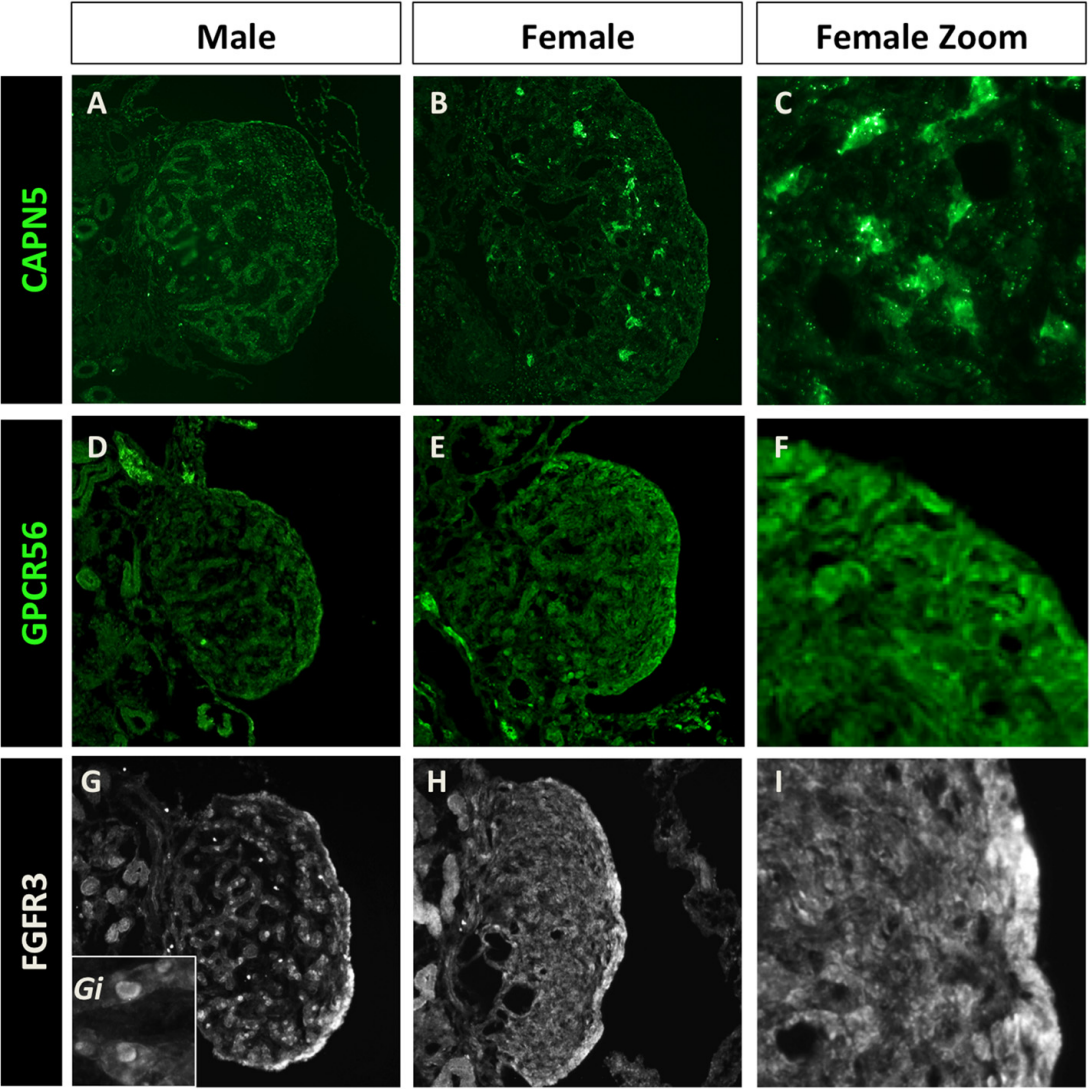
Novel candidate female genes are translated in the embryonic gonads. Immunofluorescence staining using antibodies raised in house (CAPN5 and GPR56) or commercial antibodies (FGFR3) in E8 gonads. CAPN5 protein is only weakly expressed in males (**a**), but shows high expression in a subset of cells in the female gonads (**b**). These cells are present in the juxta-cortical medulla, and staining appears cytoplasmic (**c**). GPR56 is expressed in the cortex of the female (**e**, **f**), where it appears cytoplasmic, although this staining is weak, and GPR56 is weak in male gonads (**d**). FGFR3 is expressed in the male gonads (**g**) and in the female gonads (**h**), where it is strongly expressed in the membrane or cytosol of the germ cells (see insert Gi). Strong staining in the female cortex (**i**) is probably due to germ cell expression, although may also be due to additional cortical cells expressing this receptor

To study these proteins, a commercial antibody was used for FGFR3 and in-house antibodies were raised against CAPN5 and GPR56 proteins. These new antibodies were tested for specificity (Additional file 5: Figure S2). To this end, open reading frames of chicken *CAPN5* and *GPR56* were cloned and expressed them in a chicken fibroblastic cell line (Additional file 5: Figure S2). Two polyclonal antibodies were tested for each protein, at various concentrations (data not shown). For GPR56, while low background staining was observed in untransfected DF1 cells (Additional file 5: Figure S2A), strong expression was observed when transfection with the GPR56 plasmid was carried out (Additional file 5: Figure S2B). This staining appeared to be cytoplasmic, excluded from the nucleus (Additional file 5: Figure S2Bi, Bii). CAPN5 specific staining is observed only in the transfected cells and not in the control cells (Additional file 5: Figure S2C, D), and it appears punctate throughout the cytoplasm (Di, Dii). These antibodies were then used to assay expression in embryonic gonads. CAPN5 was detected in female gonads at E6 in a subset of cells in the medulla. By E8.5 (HH stage 32) this expression had increased and was evident in a subset of juxta-cortical medullary cells (Fig. 5b). CAPN5 appeared to be cytoplasmic in these cells (Fig. 5c). This protein expression pattern was consistent with the mRNA expression pattern. Some low level punctate staining was observed in the males from E8.5 (Fig. 5a). GPR56 was also expressed from E6.5 (HH 30) in the female, with only background staining in the male. Expression of GPR56 was highest in the cortex of the ovary where it appeared to be cytoplasmic and at the plasma membrane (Fig. 5e, f). (See also Additional file 5: Figure S2E, F for additional images.) The FGFR3 antibody detected protein in the germ cells in both sexes (Fig. 5g, h), where it is cytoplasmic or at the plasma membrane (Fig. 5Gi). Staining was stronger in the ovary (Fig. 5h), especially in the cortex (Fig. 5i), which may reflect its expression in somatic cortical cells as well as germ cells.

The female-biased expression of these proteins, especially CAPN5 and GPR56, indicates that they might play a role in ovarian development from the time of sexual differentiation, and further validates RNA-seq as a useful tool for the identification of novel candidate gonad differentiation genes. Further studies including functional analysis are currently underway in our laboratory to assess the precise role of these genes.

## Discussion

Sexual differentiation of the gonads during embryonic development involves the sex-biased expression and action of several well-characterised regulators of ovarian or testicular development. However, only a small number of genes have been identified and many cases of unexplained human DSDs point to the existence of uncharacterised genetic regulators of embryonic gonadal development. RNA-seq was employed here to identify novel candidate sex genes, using embryonic chicken gonads as a model system. This study is the first to report RNA-seq in entire gonads at the time of sexual differentiation in a vertebrate species. We have combined previous analysis of female and male gonads at E4.5, prior to sexual differentiation, with new data from E6 gonads. By comparing these two time-points, a detailed view of the gonad transcriptome during sexual differentiation has been revealed.

While the expression of several known or putative gonad sex determinants has previously been assessed in embryonic chicken gonads [10, 16, 19, 60–63], this has often been based on assessment of suspected genes and pathways, employing whole mount *in situ*, qRT-PCR, or protein expression. These screens have been successful in identifying candidates, some of which are also found here (e.g., *CLDN11, INHA, InhibinA and B and BMPR2).* [64]. In addition, some of these studies have identified transcriptional differences between the left and right ovary, the latter of which regresses in chicken [64, 65]. We did not separate left and right ovary in this study. Nevertheless, these approaches are biased towards known pathways inferred from other vertebrates. They lack both the sensitivity and detail of RNA-seq. Indeed, RNA-seq can reveal early changes in gene expression undetectable by other methods.

Firstly, the RNA-seq data has provided information on the expression of known or putative sex genes. These results are interesting when considering the inferred molecular hierarchy of genes and their signalling in the gonads, both in chicken and other species. For example, in females *FOXL2* shows sexual dimorphism from E4.5, preceding that of *aromatase*, which does not become significantly DE until E6. This is consistent with previous studies that have suggested that FOXL2 acts upstream of other female genes, at least at the transcriptional level [11, 16, 66]. Indeed, in other species ranging from fishes to mammals, *FOXL2* is a putative or known activator of *aromatase* gene expression [67, 68]. Given that Aromatase expression and oestrogen synthesis is essential for ovary formation in birds, FOXL2 likely occupies a key position in the female pathway. In addition, a recent study has suggested that the main role of *FOXL2* is as a repressor of male genes [69], and this may explain the importance of early *FOXL2* expression where it would drive the gonadal primordium towards an ovarian fate by repressing male genes and activating female genes.

In the avian embryo, and indeed other vertebrates, the activator of *FOXL2* has not been clearly defined, and while a W-chromosome linked gene may fulfil this role in the chicken, no convincing candidate exists. Alternatively, FOXL2 may be activated by a factor common to both sexes that is antagonized by elevated expression of Z-linked *DMRT1*. DMRT1 can antagonise *FOXL2* expression, as evidenced in *DMRT1* knockdown gonads, which exhibit elevated *FOXL2* transcription and feminization [7]. Both Z-linked *DMRT1* and *HEMOGEN* show significant male-bias at E4.5, as does *AMH*, preceding *SOX9*, which becomes male up-regulated at E6. This indicates that DMRT1/Hemogen activate *SOX9* transcription, which is supported by recent over-expression studies *in ovo* [3, 10, 20].

As the gonads enter the phase of sexual differentiation, they up-regulate sexually dimorphic gene expression, most of which is autosomal. Given that some known sex determining genes are dimorphic prior to this activation (e.g., *DMRT1* and *FOXL2*), these activated genes at E6 (stage 29/30) are likely downstream regulators of ovarian versus testis development. Gene ontology and KEGG pathway analysis suggests that many of these genes are involved in the restructuring of the gonad into the morphologically distinct testis or ovary. There was an over-representation of genes involved in ECM and cytoskeleton formation and regulation in the datasets. Indeed, a study that used network approaches to analyse sex-biased gene expression in chickens suggested that genes of the same sex bias tended to be more connected to each other than expected, and that when genes were grouped into sex-biased modules these showed functional homogeneity [70].

Our RNA-seq was carried out on whole chicken gonads and therefore encompasses expression from both supporting cell types (presumptive granulosa and Sertoli) as well as from interstitial cells and from germ cells. To assess the relative importance of each cell type and the potential overlap with mouse gonadal expression profiles we compared our screen and three high throughput microarray screens carried out on embryonic mouse gonads/cells at a similar stage of differentiation [32–34]. We found significant enrichment (*P*-value <0.001) between the genes identified in each study and our own. In addition, these analyses indicate that we have found DE genes from not just supporting cell types, but also from the germ cell and stromal/interstitial cell pools, albeit to a lesser extent. Interestingly, we found only 76 genes were common between the three mouse studies (specifically in the supporting cells only). This probably reflects the subtle differences between each of these screens such as the transgenic mice used, the sorting methods, the microarray platforms and the analyses. Interestingly though, of these 76 genes 20 were also DE in our data set. The presence of several already characterised gonadal genes in this list means that these represent excellent candidates for further investigation.

The RNA-seq analysis also provided novel candidate genes for sexual differentiation in female and male chicken embryos. By comparing expression in females and males at E6 numerous significantly sexually dimorphic genes not previously implicated in gonad development were identified. To validate these lists, several male and female biased genes of interest were chosen, based on their gene ontology and expression. These were validated *in vivo* using *WISH* to assess their expression in the UGS and in sex reversed gonads. For all genes assayed, expression profiles in *WISH* mimicked those in the RNA-seq data, which we have also found previously with W genes [14]. For male-biased genes, expression was present in the testis cords, and was up-regulated when female gonads were experimentally sex-reversed using the aromatase inhibitor fadrozole. The female genes assayed were either present in the medulla (*CAPN5*) or the cortex (*FGFR3* and *GPR56*) of the developing ovary. In the presence of fadrozole, these female-biased genes had reduced expression, which was similar to the level observed in ZZ males. These results suggest that RNA-seq has successfully identified candidate genes for gonadal sexual differentiation in the avian system.

Only a small number of genes implicated in ovarian development have been described. Many of these have derived from analysis of human or animal disorders of sex development, i.e. WNT4 [71], R-spondin1 [72, 73], FOXL2 [74] reviewed in [1]. There are still gaps in our understanding of both the initial triggers and the downstream modulators of ovarian development. This study has identified numerous female-biased genes, which may play a role in ovarian differentiation. Indeed, we focused our studies on three of these genes, using specific antibodies against the chicken proteins. Protein expression was similar to that revealed by the RNA-seq and *WISH* analyses. Closer investigation into expression provided insight into the potential role of these genes.

One candidate gene was *FGFR3*, an FGF receptor. FGF9 plays a key role in testis differentiation, and in the regulation of germ cell development [56–58]. Several FGF receptors exist in vertebrate species. Loss of FGFR2 in mice testis leads to partial sex reversal [75], but intriguingly both *FGFR2* and *FGFR3* show significant female-biased expression in our E6 data. FGFR3 has been shown to interact with FGF1 and FGF9 [76] and is thought to be important in the post-natal testis [77]. A role for FGFR3 has not yet been described in the ovary during embryogenesis. Interestingly, *FGF9* itself was very weakly expressed in E6 gonads, and was not sex-biased, and so the role of this FGF may not be conserved outside mammals. (Although a role could be played by a related FGF). Furthermore, we did not identify any FGF ligand showing male-biased expression at E6. In contrast, *FGF16* showed robust female-biased expression. This FGF is also female-biased in Nile tilapia gonads [78], and may play a role in early oocyte development. It is interesting to speculate that FGF16 signalling though FGFR2/3 in the female may contribute to ovary differentiation or female germ cell maturation in chickens.

CAPN5 was expressed in the adrenal glands of both sexes, and the gonads of the female only, where it was found in a subset of cells present in the juxta-cortical medulla. The 3βHSD enzyme, which is involved in the synthesis of sex steroids testosterone and oestrogen is also expressed in a similar subset of cells as well as the adrenal glands (Lambeth *et al. Submitted*). This indicates that different cell lineages exist in the medulla and it appears that CAPN5 is expressed in a subset of these. These data suggest that this novel candidate may be involved in the differentiation or function of steroid producing cells in the ovary as well as in the adrenal glands of both sexes.

In contrast, our third ovarian candidate, GPR56, was expressed in cortex of the differentiating ovary as it proliferates during ovarian development. WNT4 and RSPO1 are expressed in the cortex of the developing ovary, and their activated transcription factor β-catenin appears to be stabilized there [5]. Recently, another G-protein coupled receptor, *Lgr5* has been shown to be expressed in cortical granulosa precursor cells, and plays a role in germ cell differentiation and survival [79]. Indeed, LGR5 binds R-spondin proteins and enhances RSPO/WNT/β-catenin signalling [80–83]. It will be interesting to determine if GPR56 also interacts with this pathway in the cortex of the chicken ovary.

These genes are all interesting candidates for ovarian development and functional studies including overexpression and knockdown are now underway in our laboratory to establish their exact roles. Most recently, we have developed the method of *in ovo* electroporation to deliver genes or antisense RNAs directly into developing gonads [3, 84]. This methodology is being used to manipulate the novel candidate genes identified in the current study.

Several caveats to this type of analysis that focuses on sexually dimorphic genes is that not all key genes involved in ovarian versus testicular development will be expressed in a sex-biased fashion. Some genes may be expressed in both sexes but only have a key role on one sex. In mouse embryos, *Gadd45g* and *Map3k4* are expressed in the early gonad equally in both sexes, yet mutations in these genes lead only to sex reversal in XY males [85–87] because they act on the *SRY* gene (present on the Y chromosome) through the p38 MAPK pathway. Typically these genes have been identified in human DSD cases or mouse mutants. In addition, this analysis was based on genes correctly annotated in the Galgal4 chicken genome, thus missing genes that are unannotated or lie in regions that have typically been difficult to sequence. De novo transcript assembly of the mRNAs expressed at E6 may reveal additional novel candidates for sex determination.

## Conclusions

This study represents the first RNA-seq analysis of embryonic gonads at the time of sexual differentiation. These data have provided a detailed view of the transcriptional changes that occur when sexual differentiation begins. Over 1000 genes were significantly sexually DE at this stage, most of which are autosomal genes. Many of these likely contribute to the morphological changes taking place, such as ECM remodelling and tubulogenesis. These data provide several new candidate ovary or testis differentiation genes, some of which we have validated *in vivo*. In particular, we show that CAPN5 and GPR56 are excellent candidates for ovarian development. Functional studies on these novel candidates are now underway in our laboratory, with a view to clarifying their roles in gonadal development and differentiation.

## Methods

### Embryo incubation and tissue collection

All experimental work adhered to the Australian code of practice for the care and use of animals for scientific purposes (7th edition, 2004) and to in-house animal handling guidelines (Murdoch Childrens Research Institute and CSIRO Australian Animal Health Laboratory). Ethics was from MCRI - Chicken Ethics: Institute Animal Ethics number A627. IBC (Institute Biosafety Committee): 103ext-2007, and 194–2013 EXEMPT. All experiments were carried out on a single line of Specific Pathogen Free embryos (SPAFAS) from the White Leghorn strain of chick (Lohman-LSL) and incubated at 37.8 °C with rocking in a humidified incubator. For RNA-seq, SPF eggs were incubated until stage 29 (embryonic day 6) or E4.5 see [14]. Paired gonads were removed and stored at −80 °C. Embryos were sexed by PCR as described previously [88], briefly, a small piece of limb tissue was digested in PCR compatible buffer containing proteinase K (200 μg/ml at 50 °C for at least 30 min), followed by rapid PCR amplification of the sex-linked, female-specific Xho1 sequence. Amplification of 18S ribosomal RNA genomic sequence was used as the internal control in a duplex reaction.

### RNA extraction and sequencing

Tissues were pooled according to sex. Eighteen paired gonads (i.e. left and right gonads from 18 individuals) were pooled for each replicate (two male replicates and two female replicates). Pooling has been documented as an appropriate way to prepare samples for expression analysis [89]. Total RNA was extracted using the RNeasy micro kit (QIAGEN). On column DNAsing was performed to remove contaminating genomic DNA. The resulting 4 RNA replicates were poly A-selected, reverse transcribed, fragmented, bar-coded and sequenced using the Illumina HiSeq2000 at Australian Genome Research Facility (AGRF) in Melbourne. We sequenced 100 base pair, paired ends reads.

### Bioinformatic analysis

Differential expression (DE) analysis on the raw reads was carried out by testing the female read counts against male read counts using edgeR [90], using count data from E6 and earlier time-points from [14]. *P*-values were adjusted for multiple testing using the Benjamini and Hochberg false discovery rate (FDR) method [91]. Sequenced read pairs (from both E6 and E4.5 time-points) were mapped to the chicken genome (galGal4) using the TopHat 2.0.6 software [92]. We used Ensembl version 73, which has 17108 genes. The Ensembl gene annotation was provided to TopHat to aid alignment, with other settings default. Read pairs were summarized to Ensembl gene-level counts using featureCounts [93]. Default edgeR settings were used including normalisation using TMM. Homology of the read-pairs with Ensembl genes was then calculated, and expression for each gene calculated in Fragments Per Kilobase Of Exon Per Million Fragments Mapped (FPKM).

Genes were sorted based on their minimum *P*-value (irrespective of time-point) and the top 400 DE genes were hierarchical clustered. Clustering was performed on the proportion of counts in each sample with respect to the sum across samples, after normalising for library size. The clustering and heatmap were created using the *hclust* and *heatmap.2* functions in R respectively (using default settings - agglomeration clustering using complete linkage). Gene ontology and KEGG pathway analysis were carried out using DAVID according to the default actions.

### Screen comparison

Gene lists (converted to mouse universal gene symbol) were compiled for each significantly sexually dimorphic gene (generally *P*-value <0.05, FC of 1.5 or 2) described in each study using DAVID software (from either Ensembl ID, microarray ID or Unigene ID). Gene lists were then compared using R (version 3.2.0), and one-sided Fisher exact test was used to obtain a *P*-value for enrichment.

### Embryos for in vivo studies

Fertile eggs (SPF) were obtained from CSIRO, Werribee and incubated under humid conditions at 37.8 °C. Embryos were harvested at various days throughout development and staged according to the morphological criteria of Hamburger and Hamilton [15]. The UGS including the mesonephric kidneys, gonads and Müllerian duct were dissected out in PBS and fixed for either *WISH* or immunofluorescence.

### Whole-mount in situ hybridisation

Plasmids containing fragments of mRNA for each gene were ordered from the BBSRC ChickEST Database. These were verified by PCR and sequencing. The clones were as follows; ZNF385b - ChEST470h3, BMPR2 - ChEST10191l3, NZP – ChEST203m10, Laminin - ChEST791a8 CAPN5 – ChEST992n12, FGFR3 – ChEST779m22. For the GPR56 and the ZNF385b the ORFs were also used to generate probes in pGEM T-easy. Probes were made using the either T7, Sp6 or T3 RNA polymerase and labelled with digoxygenin (DIG). In situ hybridization was performed as described previously [9, 94]. Briefly, UGS were dissected from embryos and fixed overnight at 4 °C in 4 % paraformaldehyde. At least three embryos were used for each sex and time point or condition. They were then processed for *in situ* hybridization with the DIG-labelled antisense riboprobe. For negative controls, a DIG-labelled sense riboprobe was generated for each gene. Alkaline phosphatase conjugated Anti-DIG antibodies were used, and the chromagen was BCIP/NBT. Following WISH, specimens were photographed again under bright field and were then over-stained before being imbedded in OCT for cryosectioning (sections were cut at 10, 14 and 18 μm). Staining was analysed by photography under bright field and photos taken of sections using 5x and 20x lenses. Both sense and antisense probes were initially tested for all genes. Staining was only observed with antisense probes, and not in the sense controls (data not shown).

### Immunostaining

Urogenital tissues were dissected from chicken embryos and briefly fixed for 15 min in 4 % paraformaldehyde/PBS at room temperature. Tissues were then cryoprotected by immersion in 30 % sucrose/PBS (overnight at 4 °C), infiltrated with OCT embedding compound. Ten micron frozen sections on slides were treated as previously described [63]. Secondary antibodies were AlexFluor 488 donkey anti-rabbit IgG, 1:1000, and AlexaFluor 594 donkey anti-mouse IgG, at 1:1500 from Invitrogen). Sections were mounted in Fluorosave (Calbiochem). Images were taken on a Zeiss Axiovision M1.

### Antibodies

Antibodies were raised against the chicken GPR56 and CAPN5 proteins at GenScript. Two affinity purified polyclonal rabbit antibodies were raised for each, against the peptides VSEPIDLTEGDYTTC or CHLQDRGNRRSNDLP for CAPN5 and CAFASPKEENREVQG or CWREDGTASSGNWDS, for GPR56. The FGFR3 antibody was from Bioss (bs-0165R) and used at 1:100.

### Cell culture

To test the GPR56 antibody *in vitro*, the GPR56 ORF was cloned from chicken gonad cDNA using the primers GPR56_Fow GAACCCAGGCTGAGAGCCAG, GPR56_Rev GCAAACACCCTCTGCCAGGC. Capn5_Fow GAAAGGATGTTTTCCTCA, Capn5_Rev TACACTTGCAGGTGGTAGA. GPR56 was cloned into the Tol2 plasmid under the CMV promoter. Chicken fibroblasts were transfected according to the Lipofectamine 2000 protocol (Life Technologies) with either empty or GPR56 Tol2 vector. Anti-GPR56 antibodies 1 and 2 were tested at various concentrations (1 :500, 1: 2000, 1:10,000) and assessed for specific staining using immunofluorescence as described above. CAPN5 ORF was cloned into the RCAS.BP.A avian retroviral vector. This was transfected into cells, which were passaged at least once to allow the spread of virus. These cells and control/uninfected cells were then fixed and stained with the two antibodies as above. Anti-P27 was used to check viral infection (not shown).

### Availability of supporting data

RNA-seq data for both time points has been uploaded to the DDBJ database as part of the Chicken BioProject (ref number SRP014719).

https://trace.ddbj.nig.ac.jp/DRASearch/study?acc=SRP014719.

## Additional files

Additional file 1: Table S3. Mouse screen comparison using chicken DE genes (*p*-value <0.001). Results from one-sided Fisher exact test using chicken DE genes with *p*-value 0.001 as a cutoff. (XLSX 34 kb) Additional file 2: Table S1. Genes used for DAVID analysis of gene ontology and KEGG pathways. Total genes were filtered for those showing only DE expression (*P*-value <0.001) at E6 and not E4.5, to isolate those genes that become dimorphically expressed as the gonad begin to differentiate. We removed well-characterized sex genes such as FOXL2 and SOX9 to allow identification of additionally activated pathways. This resulted in a list of 774 genes, which were subject to DAVID analysis. In the table expression (shown as FPKM) is shown for female and males at E4.5 and E6. *P*-values for the differences between females and males are shown for each time point (BH_PValue 4.5 or 6). *P*-values are adjusted using Benjamini & Hochberg method. (XLSX 193 kb) Additional file 3: Figure S1. Example of top KEGG pathways showing sex-biased expression in E6 gonads. DAVID KEGG pathway analysis was carried out on all genes showing a significant sexually dimorphic expression (*P*-value <0.001) at E6, but excluding those at E4.5 and known sex genes (Additional file 2: Table S1). This revealed several additional pathways that were significantly represented. (A) Focal adhesion pathway, which was the most highly represented KEGG pathway in DAVID analysis of all genes. Red stars indicate those genes that were in our DE list. (B) Pathway for the regulation of actin cytoskeleton. Red starts indicate genes found in our list. (TIFF 11721 kb) Additional file 4: Table S2. Top 45 female and top 45 male biased genes. For male genes we filtered for sexually dimorphic genes at E6 (*p* < 0.001) which were male biased (Fem:male ratio of less than 0), with a male gonadal expression of more than FPKM 10 at E6. These were ranked according to *P*-value. We took the top 45 genes. For female genes we filtered for sexually dimorphic genes at E6 (*p* < 0.001) which were female biased (Fem:male ratio of greater than 0), with a female gonadal expression of more than FPKM 10 at E6. In the table expression (shown as FPKM) is shown for female and males at E4.5 and E6. *P*-values for the differences between females and males are shown for each time point (BH_PValue 4.5 or 6). *P*-values are adjusted using Benjamini & Hochberg method. (XLSX 68 kb) Additional file 5: Figure S2. Validation of newly raised antibodies for chicken GPR56 and CAPN5. Antibodies were tested at various concentrations (not shown) in chicken DF1 fibroblasts either transfected with a control plasmid or a plasmid expression protein of choice. (A) Only low background staining is shown for GPR56 in control cells, whereas when transfected with GPR56 expressing plasmid staining was observed (B). This staining is mostly cytoplasmid (see *Bi, Bii*). (C) Some weak background staining was seen in control cells, compared to those over-expressing CAPN5 (D). This staining is punctate and cytoplasmic (*Di, Dii*), similar to what is seen *in vivo*. Additional examples of *in vivo* staining for GPR56 in male E8.5 gonads (E) and female (F). (TIFF 11721 kb)

## Abbreviations

DSDs: Disorders of sex development
HH: Hamburger and Hamilton stage
E: Embryonic day
RNA-seq: RNA sequencing
GFP: Green fluorescent protein
ECM: Extracellular matrix
DE: Differential expression
FPKM: Fragments Per Kilobase of Exon Per Million Fragments Mapped
FDR: False discovery rate
BP: Biological process
CC: Cellular component
MF: Molecular function
KEGG: Kyoto Encyclopedia of Genes and Genomes
UGS: Urogenital system
SPAFAS: Specific Pathogen Free embryos
FC: Fold change
