# Erratum to: ‘Identification of candidate gonadal sex differentiation genes in the chicken embryo using RNA-seq’

**DOI:** 10.1186/s12864-016-2439-2

**Published:** 2016-03-02

**Authors:** Katie L. Ayers, Luke S. Lambeth, Nadia M. Davidson, Andrew H. Sinclair, Alicia Oshlack, Craig A. Smith

**Affiliations:** Murdoch Childrens Research Institute, Royal Children’s Hospital, Flemington Road, 3052 Parkville, VIC Australia; Department of Paediatrics, University of Melbourne, Parkville, VIC Australia; Department of Anatomy and Developmental Biology, Monash University, Clayton, VIC 3168 Australia

Unfortunately, the original version of this article [1] contained an error in Figure 2. The correct figure is included below:

**Fig. 2:**
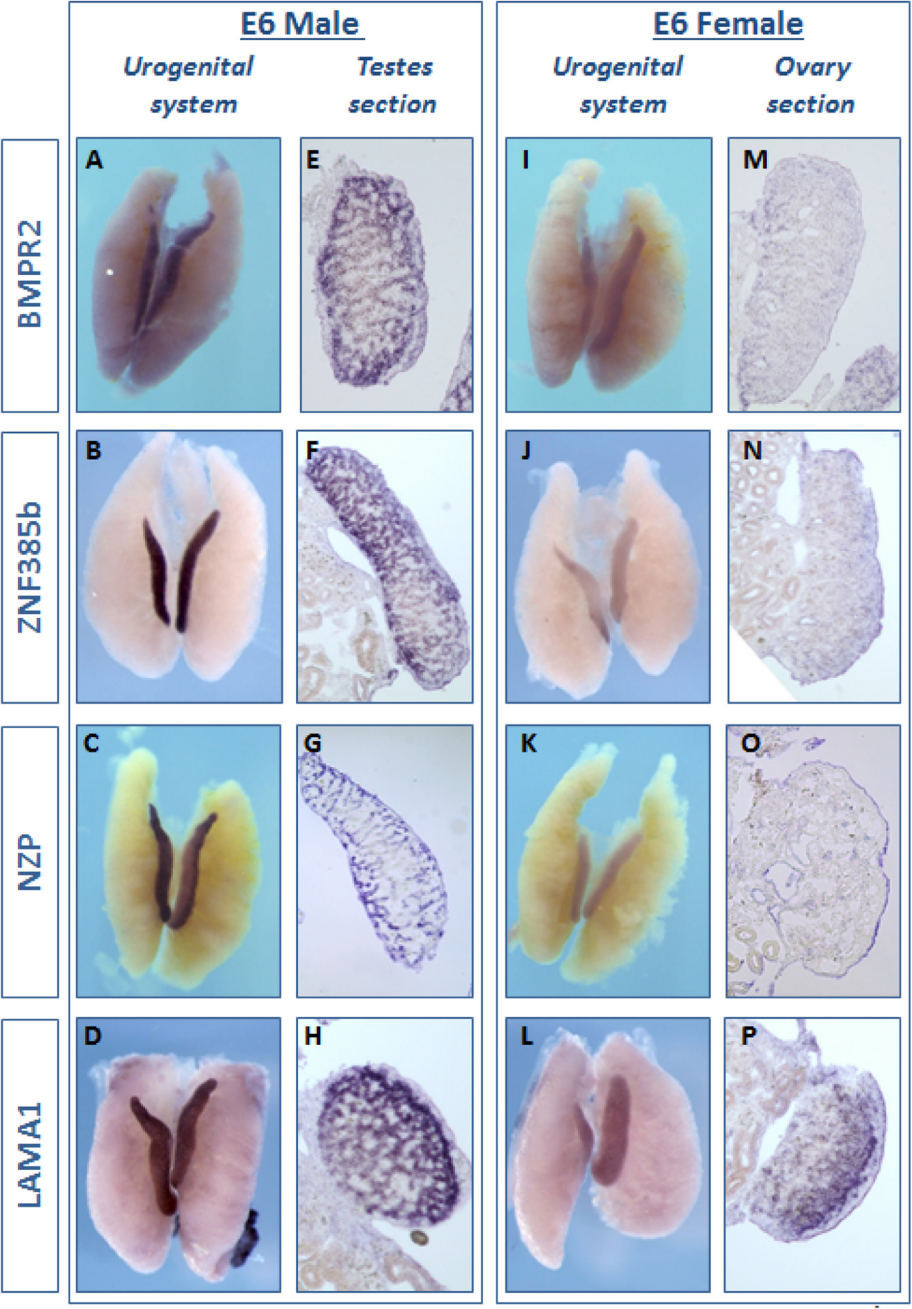
Male candidate gene expression *in vivo.* Whole mount *in situ* hybridisation for 4 male-biased candidate genes, on E6 UGS (UGS) from males and females. *BMPR2* is more highly expressed in males (**a**, **e**) than in females (**i**, **m**). In over-stained sections, *BMPR2* appears to be expressed in the testis cords (**e**). *ZNF385b* shows greater expression in males (**b**, **f**) than females (**j**, **n**), consistent with RNA-seq. In males it is expressed in the cords (**f**). *NZP*, a novel Z-protein, is expressed in males more highly than females (**c**, **g** versus **k**, **o**). It is also expressed in the cords of males (**g**). *LAMA1* is also higher in males (**d**) than females (**l**) and is expressed in testis cords in males (**h**), with some weak expression in the juxta-cortical medulla in females (**p**). These results are consistent with RNA-seq data. Typically, 3 UGS from each sex were used for each probe, and these images are representative. A sense control probe did not show any staining for any of the candidate genes (data not shown)

## References

[1] Ayers KL, Lambeth LS, Davidson NM (2015). Identification of candidate gonadal sex differentiation genes in the chicken embryo using RNA-seq’. BMC Genomics.

