# Supplementary figures and images for: Identification of candidate gonadal sex differentiation genes in the chicken embryo using RNA-seq
Source: BMC Genomics. 2015 Sep 16;16(1):704. doi: 10.1186/s12864-015-1886-5 (PMC4574023; doi:10.1186/s12864-015-1886-5)

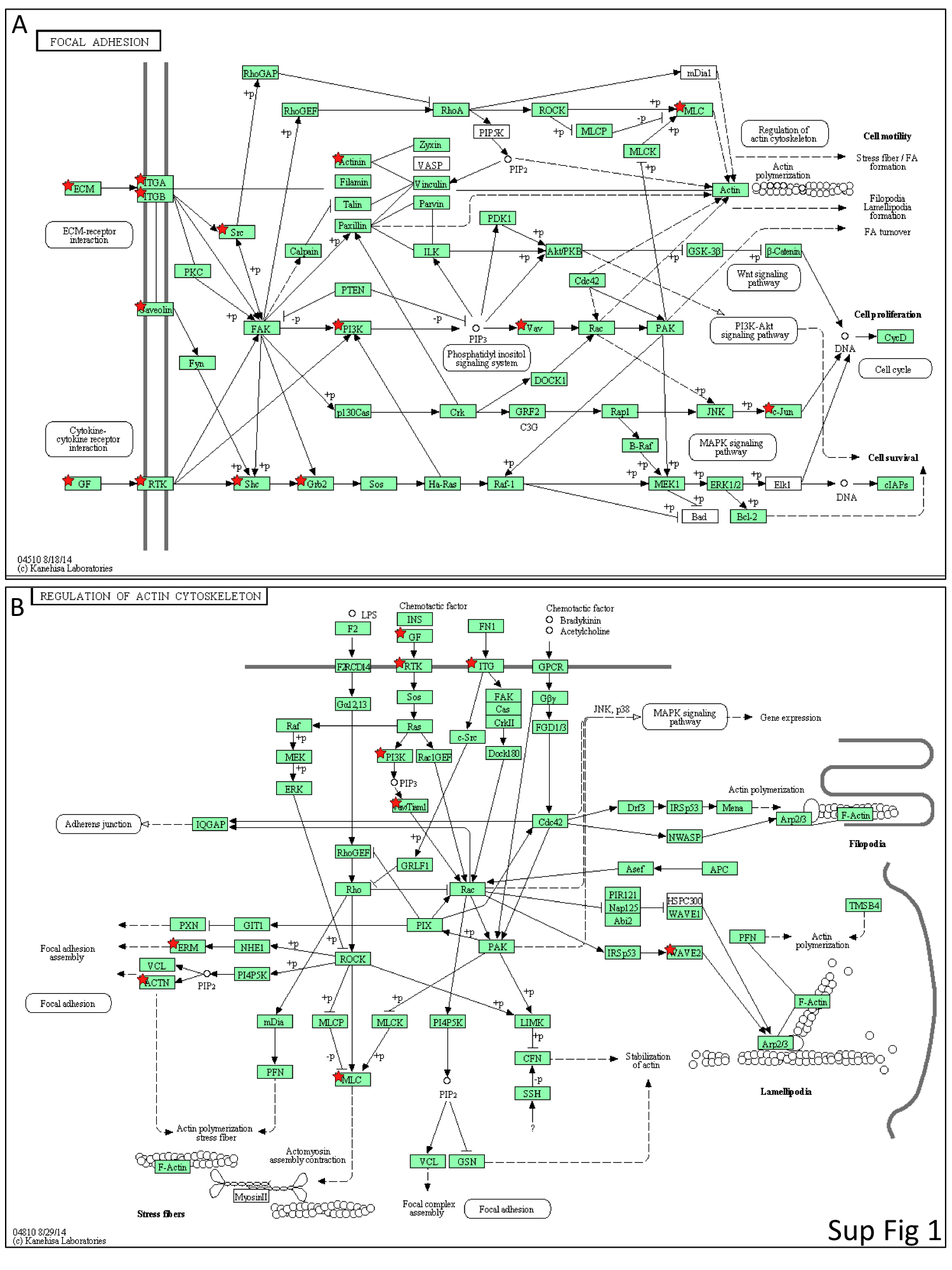

Supplement: Additional file 3: Figure S1. — Example of top KEGG pathways showing sex-biased expression in E6 gonads. DAVID KEGG pathway analysis was carried out on all genes showing a significant sexually dimorphic expression (P-value <0.001) at E6, but excluding those at E4.5 and known sex genes (Additional file 2: Table S1). This revealed several additional pathways that were significantly represented. (A) Focal adhesion pathway, which was the most highly represented KEGG pathway in DAVID analysis of all genes. Red stars indicate those genes that were in our DE list. (B) Pathway for the regulation of actin cytoskeleton. Red starts indicate genes found in our list. (TIFF 11721 kb) [file 12864_2015_1886_MOESM3_ESM.tiff]

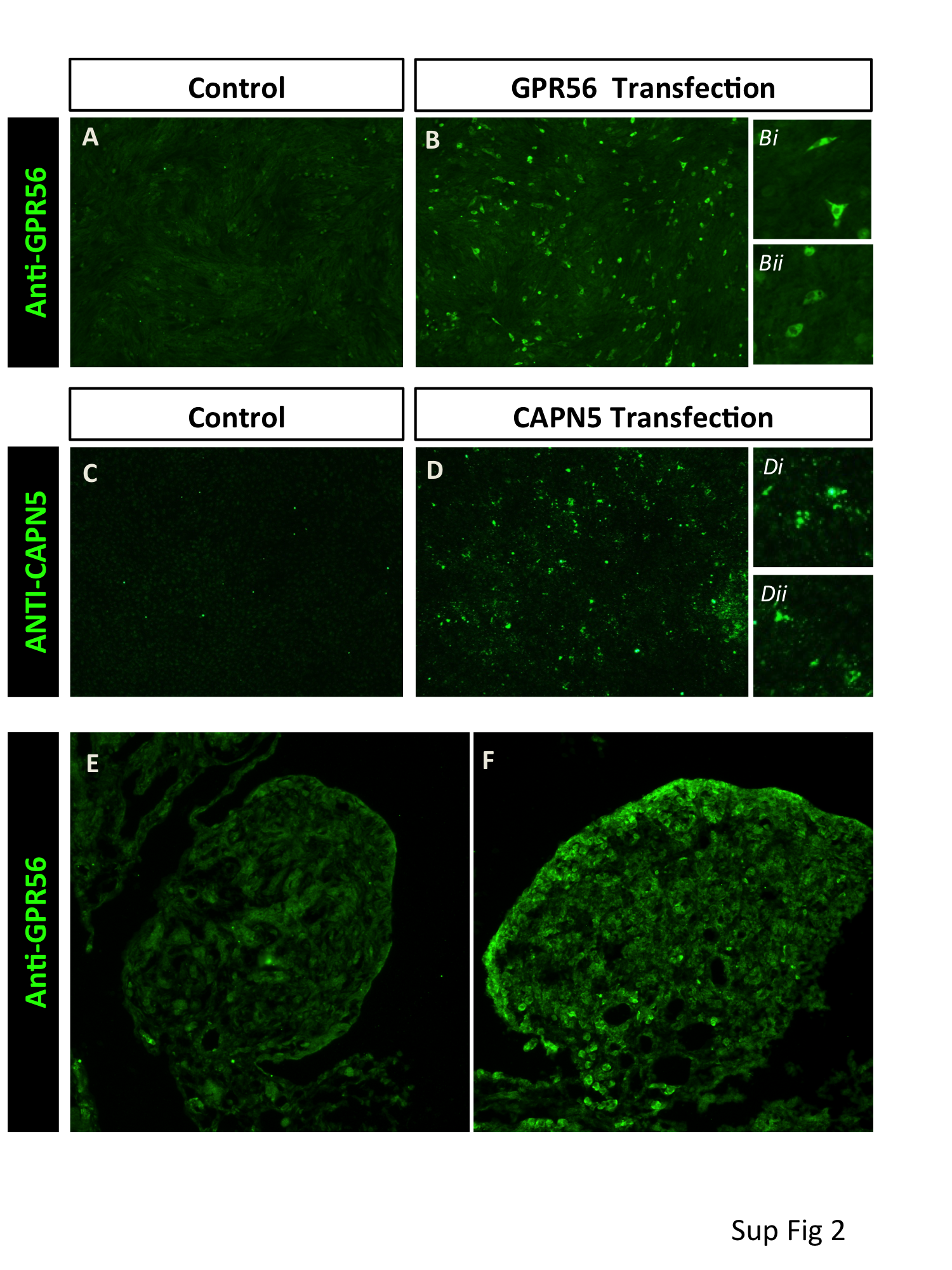

Supplement: Additional file 5: Figure S2. — Validation of newly raised antibodies for chicken GPR56 and CAPN5. Antibodies were tested at various concentrations (not shown) in chicken DF1 fibroblasts either transfected with a control plasmid or a plasmid expression protein of choice. (A) Only low background staining is shown for GPR56 in control cells, whereas when transfected with GPR56 expressing plasmid staining was observed (B). This staining is mostly cytoplasmid (see Bi, Bii). (C) Some weak background staining was seen in control cells, compared to those over-expressing CAPN5 (D). This staining is punctate and cytoplasmic (Di, Dii), similar to what is seen in vivo. Additional examples of in vivo staining for GPR56 in male E8.5 gonads (E) and female (F). (TIFF 11721 kb) [file 12864_2015_1886_MOESM5_ESM.tiff]
